# Vitamin D: Bolus Is Bogus—A Narrative Review

**DOI:** 10.1002/jbm4.10567

**Published:** 2021-10-30

**Authors:** Richard B. Mazess, Heike A. Bischoff‐Ferrari, Bess Dawson‐Hughes

**Affiliations:** ^1^ Department of Medical Physics University of Wisconsin Madison WI USA; ^2^ Department of Aging Medicine and Aging Research University of Zurich Zurich Switzerland; ^3^ City Hospital Zurich University Clinic for Aging Medicine Zurich Switzerland; ^4^ Jean Mayer US Department of Agriculture (USDA) Human Nutrition Research Center on Aging Tufts University Boston MA USA

**Keywords:** PTH/VIT D/FGF23, CELL/TISSUE SIGNALING, ENDOCRINE PATHWAYS, CLINICAL TRIALS, NUTRITION, AGING

## Abstract

In this review we summarize the impact of bolus versus daily dosing of vitamin D on 25(OH)D and 1,25(OH)_2_D levels, as well as on key countervailing factors that block vitamin D functions at the cellular level. Further, we discuss the role of bolus versus daily dosing of vitamin D for several health outcomes, including respiratory infections and coronavirus disease 2019 (COVID‐19), rickets, falls and fractures, any cancer, and cancer‐related mortality. This discussion appears timely because bolus doses continue to be tested for various disease outcomes despite a growing amount of evidence suggesting lack of efficacy or even detrimental effects of bolus dosing of vitamin D for outcomes where daily dosing at modest levels was effective in the vitamin D deficient. As a result, these discordant results may bias health recommendations for vitamin D if the recommendations are based on meta‐analyses combining both daily and bolus dosing trials. © 2021 The Authors. *JBMR Plus* published by Wiley Periodicals LLC on behalf of American Society for Bone and Mineral Research.

## Introduction

Fifty years ago, the metabolism of vitamin D became elucidated in detail allowing clarification of its role in the skeletal system and stimulating examination of its extraskeletal effects.^(^ ^1^ ^)^ It was not until 30 years later that its role in systemic immunomodulation was elaborated and only 10 years ago did its role in cellular immune responses, autocrine and paracrine, unfold.^(^ ^2^, ^3^ ^)^ That intracellular process, mediated by both cholecalciferol, or D_3_, and the small free portion of calcifediol (25(OH)D) rather than circulating calcitriol (1,25(OH)_2_D), accounts for over 90% of the innate and adaptive immune response of vitamin D.^(^ ^4^, ^5^ ^)^ This tightly regulated relationship of enzymatic transformation depends on cytochrome P450 2R1 (CYP2R1) for initial hydroxylation and then on cytochrome p450 27B1 (CYP27B1) for further hydroxylation to 1,25(OH)_2_D, versus enzymatic destruction of 1,25(OH)_2_D by cytochrome P450 family 24 subfamily A member (CYP24A1) 24‐hydroxylation.^(^ ^3^ ^)^

Over the past 20 years there have been many clinical trials examining supplementation of vitamin D in disease prevention. The low cost and safety of vitamin D often has led to its evaluation in the general public rather than in a cohort of deficient subjects with specific risk of a disease. As a consequence, much of the supplementation research is compromised, hence the value of correcting deficiencies remains uncertain based on the results of meta‐analyses that include these trials. There are also other reasons why trials may not have shown clear effects of supplementation on disease outcomes,^(^ ^6^, ^7^ ^)^ as shown in Table 1.

**Table 1:** Factors Potentially Affecting Vitamin D Supplement Trial Outcomes

(a) Subjects with mixed or replete baseline 25(OH)D levels rather than deficiency or insufficiency (b) Results affected by obesity and aging (c) Discordant increases of blood levels (d) Short‐term focus when longer‐term intervention is needed (e) Failure to consider baseline level and achieved level of 25(OH)D (f) Use of large bolus doses rather than daily or weekly dosing at modest levels

In this review, we explore whether bolus dosing may be a design feature that leads to variable results of trials with several different outcomes. Our focus on bolus dosing arose because its lack of success was recently shown in several areas, including meta‐analyses of acute respiratory infection trials,^(^ ^8^, ^9^ ^)^ in contrast to some studies in the same areas with positive results using daily or weekly dosing.^(^ ^8^, ^9^ ^)^ It has long been known that high vitamin D dosing turns off the tightly regulated hormonal activation process of vitamin D, in both renal‐skeletal and extrarenal (intracrine) metabolism, thereby inhibiting its modulatory function for weeks or longer. In fact, large bolus doses trigger countervailing factors, such as 24‐hydroxylase (CYP24A1), that results in downregulation of 1,25(OH)_2_D.^(^ ^3^ ^)^ In contrast, moderate dosing at short intervals (daily or every other day) is preferred because it does not trigger countervailing factors. Additionally, cholecalciferol itself, with a 20‐hour half‐life, is active intracellularly.^(^ ^3^, ^10^ ^)^ Moreover, as suggested by Hollis and Wagner,^(^ ^11^ ^)^ most cells have 25‐hydroxylase activity and therefore can utilize cholecalciferol directly, whereas 99% of 25(OH)D is bound (or deposited in fat) and not available to cellular use. Further, cholecalciferol itself has been found to have greater cellular effects than either 25(OH)D or 1,25(OH)_2_D.^(^ ^12^ ^)^

## Effects of Bolus Versus Daily Dosing on Countervailing Factors

Several studies tested the effect of vitamin D dose on the resulting change in 25(OH)D concentrations.^(^ ^13^ ^)^ The circulating 25(OH)D level has been chosen as the usual marker of vitamin D status because of its relatively long half‐life of 20 days and bolus dosing with vitamin D dramatically increases circulating 25(OH)D levels. In fact, a meta‐analysis of 30 studies using bolus dosing found that doses of >100,000 IU increased 25(OH)D levels significantly, with levels peaking between 7 and 30 days.^(^ ^14^ ^)^ However, this is a limited short‐term effect and the longer‐term systemic effects on 1,25(OH)_2_D and the upregulation of countervailing factors, such as 24‐hydroxylase (CYP24A1), that results in downregulation of 1,25(OH)_2_D, were not studied. Also, the effects of bolus dosing on antimicrobial proteins (cathelicidin [LL‐37]), defensins, or regulatory T cells were not examined. Notably, unlike daily doses of D_3_, large bolus doses have failed to increase anti‐inflammatory cytokines or decrease C‐reactive protein response.^(^ ^14^ ^)^ However, bolus doses of vitamin D almost double the risk of hypercalcemia.^(^ ^15^ ^)^

Regarding the direct treatment with calcifediol, it has been shown to increase the blood level of 25(OH)D faster than cholecalciferol by a factor of 3.2.^(^ ^16^ ^)^ This, however, may not indicate greater efficacy in 1,25(OH)_2_D production and immunomodulation. Notably, in a direct comparison of daily calcifediol dosing (5 μg, 10 μg, or 15 μg) versus 20 μg of D_3_ daily over 24 weeks in four groups of women (*n* = 59) over 65 years of age, 20 μg of D_3_ produced a blood level of 25(OH)D that was 20% lower than that produced by 10 μg of calcifediol (2.5× less potent).^(^ ^17^ ^)^ However, the 1,25(OH)_2_D produced with 20 μg of D_3_ was equal to that of 15 μg of calcifediol, and the production of the countervailing factor 24,25(OH)_2_D_3_ with D_3_ was only about half that of 15 μg of calcifediol. The increases in 25(OH)D correlated highly (*r* = 0.91) with those of 24,25(OH)_2_D_3_ but not with 1,25(OH)_2_D_3_. Another comparison of calcifediol and cholecalciferol, each at 20 μg/day, over 14 weeks in postmenopausal women (mean 61.5 years) showed that the doubled 25(OH)D response of calcifediol was not accompanied by a difference of 1,25(OH)_2_D, and five of seven markers of innate immune function declined with both.^(^ ^18^ ^)^ One comparative study over 7 months in older adults (>75 years with 90% deficient) using equal doses (150 μg/week) showed calcifediol produced only a 20% higher serum level of 25(OH)D.^(^ ^19^ ^)^ Both were equal in doubling 1,25(OH)_2_D and reducing parathyroid hormone (PTH) and C‐reactive protein (CRP). Thus, increases of 25(OH)D with bolus doses of vitamin D or with calcifediol can be deceptive because they do not increase 1,25(OH)_2_D as much as supposed.

**Fig 1:**
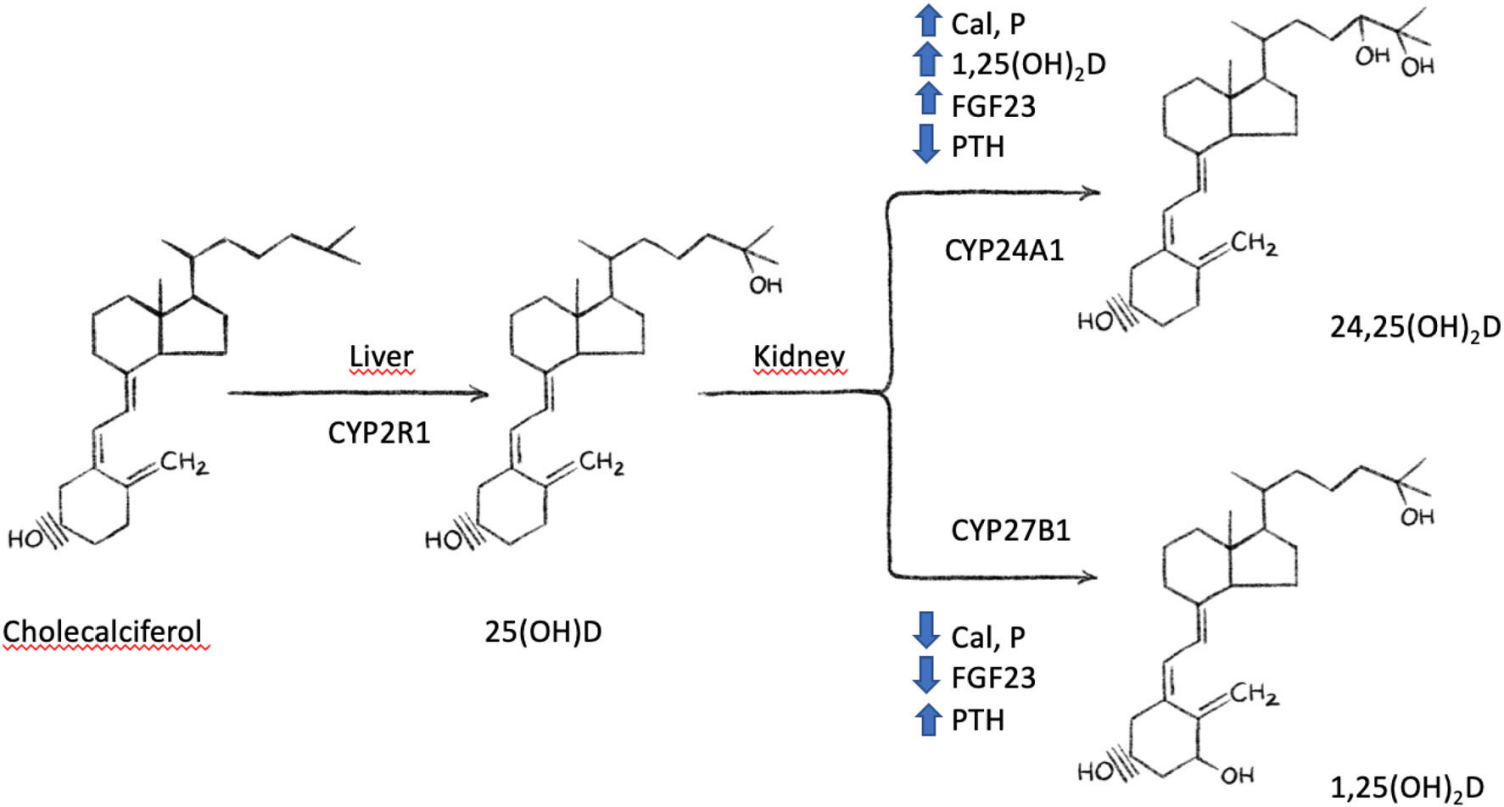
Vitamin D metabolism.

Also, there is a concern that markedly elevated 25(OH)D levels disrupt the tightly regulated production of 1,25(OH)_2_D by stimulating fibroblast growth factor 23 (FGF23), which in turn suppresses 1α‐hydroxylase (CYP2R1).^(^ ^20^ ^)^ Additionally, markedly elevated 25(OH)D levels activate 24‐hydroxylase (CYP24A1), which converts 1,25(OH)_2_D into an inactive form (see Fig. 1). These factors affect both renal and extrarenal (intracrine) metabolism. In contrast, a meta‐analysis of 23 studies, of which 14 were in end‐stage renal disease, concluded that FGF23 was not increased with daily vitamin D supplementation <2000 IU, and if the 25(OH)D level was under <40 ng/mL.^(^ ^21^ ^)^ A note of caution is appropriate when comparing FGF23 levels in trials that used different FGF23 assays, because some assays measure intact FGF23 and others measure a combination of the intact molecule and an inactive C‐terminal fragment.

The trigger of countervailing factors as a response to bolus dosing of D_3_ is apparent even at weekly intervals. Owens and colleagues^(^ ^22^ ^)^ tested 35,000 IU and 70,000 IU of D_3_ as a weekly bolus to athletes for 12 weeks and then followed them for an additional 6 weeks. At week 12, the increase with the higher dose was only 10% to 15% higher than that with the lower dose, but at week 12 the higher dose caused a 2.4× increase of 24,25(OH)_2_D compared to a 0.4× increase at the lower dose.^(^ ^22^ ^)^ Consequently, there was a similar (about 20%–30%) increase of 1,25(OH)_2_D with both doses, and serum 1,25(OH)_2_D declined to baselines in both groups at week 18. Notably, the elevated 24,25(OH)_2_D persisted for 6 weeks after dosing ended, reflecting the longer‐term induction of 24‐hydroxylase blocking the formation of 1,25(OH)_2_D.^(^ ^22^ ^)^ Fassio and colleagues^(^ ^23^ ^)^ gave healthy adults under age 60 years three different treatment regimens of vitamin D including daily and bolus dosing concepts (group A: 10,000 IU/d for 8 weeks followed by 1000 IU/d for 4 weeks; group B: 50,000 IU/week for 12 weeks, group C: 100,000 IU every other week for 12 weeks), amounting to the same cumulative dose of 600,000 IU D_3_, over 12 weeks.^(^ ^23^ ^)^ They reported that daily dosing was superior in elevating 25(OH)D with a 20% higher area under the curve. In another study, a single bolus of 150,000 IU compared to the same amount by daily dosing (5000 IU) over 30 days, produced somewhat higher 25(OH)D concentrations over the first 15 days in non‐deficient women.^(^ ^24^ ^)^ However, the countervailing factor 24,25(OH)_2_D_3_ increased about 50% with the bolus and 30% with daily dosing.

## Effects of Vitamin D Dosing on Acute Respiratory Infections and Coronavirus Disease 2019

Mechanistically, vitamin D is relevant to healthy lungs because the alveolar epithelium has been found to convert it locally into active 1,25(OH)_2_D.^(^ ^10^ ^)^ This is further supported by the well‐established effects of vitamin D on the innate and adaptive immune response,^(^ ^4^, ^25^ ^)^ including reduction of inflammation and the inhibition of cytokine excess by regulatory T cells (Tregs).^(^ ^26^ ^)^ Also, the cathelicidin LL‐37 produced by vitamin D causes apoptosis of infected airway epithelium.^(^ ^27^ ^)^

In a cohort of 9548 patients followed over 15 years, mortality from respiratory disease was twofold higher in those with 25(OH)D levels <20 ng/mL and threefold higher with levels <12 ng/mL.^(^ ^28^ ^)^ However, prospective studies of supplementation have yielded conflicting results. The New Zealand Vitamin D Assessment (ViDA) study (*n* = 5111) that used a monthly bolus dose of 100,000 IU found no benefit for respiratory infections over 3 years even for those with baseline deficiency <20 ng/mL.^(^ ^29^ ^)^ An expanded study (*n* = 21,315) providing a monthly bolus of 60,000 IU to adults aged 60 to 79 years over 5 years found no reduction of infections compared to placebo although the duration and severity of symptoms was decreased.^(^ ^30^, ^31^ ^)^ A clarification of those findings was provided by Martineau and colleagues,^(^ ^8^ ^)^ who evaluated individual participant data on about 11,000 participants from a previously reported meta‐analysis of 25 vitamin D trials for acute respiratory infection. Bolus dosing was used in 10 of the trials (*n* = 5595), whereas 15 trials (*n* = 5133) used daily or weekly doses. A 20% reduction of risk was shown for daily or weekly D_3_, but not in those receiving one or more bolus doses. That protective value of daily or weekly dosing was greatest (70%) in those with initial deficiency (25(OH)D < 10 ng/mL) versus those with insufficiency (25%). Heterogeneity among trials was due in large part to bolus dosing and the degree of initial deficiency. An expanded meta‐analysis^(^ ^9^ ^)^ identified 46 randomized controlled trials (RCTs) including 75,541 participants aged 0 to 95 years, but unfortunately the researchers did not obtain individual participant data that would have allowed accurate assessment of factors affecting responses. Vitamin D compared with placebo slightly (8%) reduced respiratory infections; bolus doses had no benefit, whereas daily dosing reduced infections by 22%.^(^ ^9^ ^)^ A similar discordance due to a preponderance of bolus dosing has been observed in meta‐analyses of D_3_ in chronic obstructive pulmonary disease (COPD) patients.^(^ ^32^ ^)^

The beginning of the coronavirus disease 2019 (COVID‐19) pandemic in 2020 led to observations that the lungs are the primary infection site of COVID with secondary vascular manifestations.^(^ ^33^ ^)^ Early observations of increased COVID exacerbation in adults with vitamin D deficiency soon led to suggestions that supplementation with vitamin D needs investigation.^(^ ^30^, ^34^, ^35^ ^)^ This was further supported by an association between 25(OH)D level and the degree of regional and total lung involvement observed in COVID patients,^(^ ^36^, ^37^ ^)^ as well as an association between the hyperinflammatory response or cytokine storm, characteristic in COVID‐19 infection, produced by lung macrophages.^(^ ^25^, ^38^ ^)^

As the pandemic continued several observational studies suggested that 25(OH)D insufficiency (<20 ng/mL) slightly increased risk of COVID‐19 infection in adults^(^ ^39^, ^40^, ^41^ ^)^; one aberrant study using decade‐old 25(OH)D results and inappropriate statistical adjustments found no association.^(^ ^42^ ^)^ However, the most recent meta‐analysis of cohort studies on the topic showed an 80% increased risk of infection with vitamin D deficiency in 14 studies.^(^ ^43^ ^)^ Other observational studies suggested that hospitalization rates are doubled, and critical care and mortality are tripled in adults with vitamin D deficiency.^(^ ^30^, ^44^, ^45^ ^)^ Further, Akbar et al^(^ ^46^, ^47^ ^)^ reviewed 14 studies with almost one million adults in 2021 and concluded that 25(OH)D deficiency was associated with both severity of COVID and threefold higher odds of mortality. This association was confirmed by another recent review supporting a twofold increased risk of mortality and a fourfold increased risk of critical care admission, although the authors pointed out the weak quality of observational studies.^(^ ^48^ ^)^

RCTs of vitamin D treatment in COVID patients are ongoing; only four trials have been reported. A Brazilian trial that gave a single bolus of 200,000 IU to 120 patients hospitalized for COVID‐19 found it of no benefit on length of stay or mortality compared to 120 controls.^(^ ^49^ ^)^ This is consistent with the results of the meta‐analyses showing that bolus dosing did not reduce acute respiratory infections, as well as length of stay, severity, or mortality in critically ill patients.^(^ ^8^, ^9^ ^)^ A different approach was taken in three Spanish trials. Entrenas Castillo and colleagues^(^ ^50^ ^)^ treated 50 of 76 newly hospitalized COVID‐19 patients with oral calcifediol (532 μg) or no calcifediol upon admission, next to the same standard of care. Patients in the calcifediol treatment group continued with oral calcifediol (266 μg) on days 3 and 7, and then weekly until discharge or intensive care unit (ICU) admission. Only one treated patient required ICU admission (2%), whereas 13 of 26 (50%; *p* < 0.001) patients on standard non‐calcifediol treatment required ICU admission.^(^ ^50^ ^)^ A second trial tested the impact of 532 μg of calcifediol initially followed by 266 μg on days 3, 7, 15, and 30 in newly hospitalized COVID patients.^(^ ^51^ ^)^ Of the 447 patients treated with calcifediol at admission, 20 (4.5%) required ICU, whereas 82 (21%) of 391 nontreated COVID patients required ICU (*p* < 0.0001). There was also a difference in mortality, 21 (4.7%) out of 447 patients treated with calcifediol compared to 62 patients (15.9%) out of 391 nontreated dying (*p* = 0.0001). The ongoing nationwide US Vitamin D for COVID‐19 (VIVID) trial is a pragmatic, cluster randomized, double‐blinded trial enrolling 1500 newly diagnosed individuals with COVID‐19 infection and will test 3200 IU vitamin D/d versus placebo (Table 2).^(^ ^52^ ^)^

**Table 2:** RCT Data Regarding Acute Respiratory Infections and COVID‐19

Studies	RCTs bolus dosing	RCTs daily dosing
Acute respiratory infections—meta‐analyses		
Martineau and colleagues^(^ ^8^ ^)^ meta‐analysis 2019 (individual participants data from 25 RCTs)	No benefit	20% Reduction with daily or weekly D3 70% reduction among those with 25(OH)D levels <10 ng/mL
Jolliffe and colleagues^(^ ^9^ ^)^ meta‐analysis 2021 (46 RCTs)	No benefit	22% Reduction with daily dosing D3
COVID‐19 single RCTs		
Murai and colleagues^(^ ^49^ ^)^ RCT with 120 participants (bolus 200,000 IU)	No benefit	
Entrenas Castillo and colleagues^(^ ^50^ ^)^ RCT treated 50 of 76 newly hospitalized COVID‐19 patients with oral calcifediol (532 μg) or no calcifediol upon admission, and oral calcifediol (266 μg) on day 3 and 7, and then weekly until discharge or ICU admission		Reduction in ICU admission Only one treated patient required ICU admission (2%), whereas 13/26 (50%; *p* < 0.001) patients on standard non‐calcifediol treatment required ICU admission
Nogues and colleagues^(^ ^51^ ^)^ RCT treated 447 of 838 newly hospitalized COVID patients with 532 μg of calcifediol initially followed by 266 μg on days 3, 7, 15, and 30		Reduction in ICU admission and mortality Of the 447 patients treated with calcifediol at admission, 20 (4.5%) required ICU, whereas 82/391 (21%) nontreated required ICU (*p* < 0.0001) There was also a difference in mortality, 21/447 (4.7%) patients treated with calcifediol compared to 62/391 patients (15.9%) nontreated dying (*p* = 0.0001)

## Effects of Bolus Dosing on Rickets

Treatment with daily low‐dose vitamin D (400 IU), and/or calcium, has been the standard for prevention of rickets in nutritionally deprived children.^(^ ^1^ ^)^ Although several small, short‐term studies have shown some success with bolus doses, both intramuscular and oral, for rickets, the evidence for their efficacy in a recent Cochrane review was inconclusive.^(^ ^53^ ^)^ A vitamin D supplementation study of 3060 stunted children under age 1 year was done in Afghanistan. Half were on placebo and half received 100,000 IU of vitamin D every 3 months for 18 months.^(^ ^46^ ^)^ There was no effect of the bolus vitamin D treatment on growth, and in a subgroup of 20% of children who received radiographic evaluation, rickets frequency was the same (5.4%) between bolus vitamin D and placebo groups. Clearly bolus dosing of vitamin D also contrasts sharply with the historical precedent of daily dosing benefits in the prevention and treatment of rickets.^(^ ^1^ ^)^ This was an issue leading several experts to advocate medical use of daily dosing rather than boluses.^(^ ^54^ ^)^

## Effects of Bolus Dosing on Falls and Fractures

Observational studies show that vitamin D deficiency is common in the sarcopenia and muscular atrophy among older adults as well as in osteoporosis and osteomalacia.^(^ ^1^ ^)^ The consequences of deficiency when combined result in the high rate of falls and fractures in the elderly. Despite this, supplementation trials with vitamin D, and its active analogs, have produced widely discordant findings. For example, a review of observational studies concluded that each 10 ng/mL increase in 25(OH)D concentration was associated with a 20% reduction of femoral fracture but no benefit was shown in the accompanying review of RCTs.^(^ ^55^ ^)^ Some reasons for this anomaly are that the trials have employed a wide variation of subjects, not just frail elderly fallers with both low baseline 25(OH)D levels and low bone density. Researchers at times are confusing vitamin D supplementation for public health with trials to resolve a clinical issue. Trials also have shown wide variation of: (i) daily supplementation doses, (ii) dose and interval of boluses, (iii) exclusion of even modest calcium co‐administration, (iv) observation length, as well as (v) disregard for levels of achieved 25(OH)D. Moreover, the underlying mechanisms involved in falls (muscle strength, neuromuscular control, and balance) differ from those fundamental in fracture (bone density). There obviously will be subsets of a population in which each of these factors dominate, for example aging bone loss in males versus females, in addition to the usual factors affecting vitamin D deficiency (diet, obesity, aging, insolation, renal compromise). Cohesive analysis from multiple studies therefore requires pooling of individual participant data. Meta‐analysis, with its usual weighting approach and forest plots, is deceptive, as shown by several recent meta‐analyses that concluded there was no effect of vitamin D supplementation on fracture risk.^(^ ^56^, ^57^, ^58^ ^)^ Some fundamental defects of such specious meta‐analyses were outlined by Heaney^(^ ^59^ ^)^ a decade ago.

Studies of sarcopenia, muscle strength, and falls have demonstrated a complex response to supplementation. Trials in the elderly with deficiency have shown that low doses have little effect on muscle strength or lower extremity function, but modest daily dosing (1000 to 3000 IU) increased strength.^(^ ^60^, ^61^ ^)^ Several meta‐analyses of trials in older adults with deficiency indicate a reduction in falls with modest daily dosing of D_3_ supplementation, although conclusions have varied by dose and target population.^(^ ^62^, ^63^, ^64^, ^65^, ^66^, ^67^, ^68^, ^69^, ^70^ ^)^ Overall, low‐dose D_3_ (<700 IU/d) did not reduce fall risk significantly,^(^ ^63^ ^)^ a reduction was seen with daily D_3_ of 700 to 1000 IU vitamin D,^(^ ^63^ ^)^ but large bolus dose (monthly 60,000 to 100,000 IU of vitamin D or annual dosing of 300,000 IU to 500,000 IU) increased fall risk among frail older adults. For example, long‐term care residents receiving 100,000 IU/month for 1 year doubled falls, but not fractures, even though 25(OH)D increased only from 25 to 32 ng/mL.^(^ ^71^ ^)^ On the other hand, a lower bolus overload (150,000 IU every 3 months) produced a similar increase of 25(OH)D concentrations without affecting falls or muscular function.^(^ ^72^ ^)^ Daily dosing at an equivalent level in replete older subjects does not increase falls. The Vitamin D and Omega‐3 Trial (VITAL) found that supplementation with 2000 IU/d in 25,871 healthy and replete (mean 31 ng/mL) adults over age 50 years resulted in a fall rate over 5 years that was identical to the fall rate of controls.^(^ ^73^ ^)^ Similarly, the VitaminD3‐Omega3‐Home Exercise‐Healthy Ageing and Longevity Trial (DO‐HEALTH) showed that 2000 IU/d over 3 years, producing a 25(OH)D increase from 24 to 38 ng/mL, did not affect leg function or nonvertebral fractures in mostly replete subjects.^(^ ^74^ ^)^

Falls and fractures are more common in older women than men, the latter being associated with the greater aging bone loss of females. This discordant pattern in falls complicates the evaluation of vitamin D on resultant fractures, but the adverse effect of bolus dosing on falls does seem to be reflected in fracture risk. For example, Sanders and colleagues^(^ ^75^ ^)^ tested a D_3_ bolus of 500,000 IU annually compared with placebo for up to 5 years among 2256 women aged 70 years and older who were at risk of fracture.^(^ ^75^ ^)^ Their initial mean 25(OH)D level was 20 ng/mL.^(^ ^74^, ^75^ ^)^ and fracture and fall rates were, respectively, 26% and 15% higher in the treated group. A study using 300,000 IU intramuscular vitamin D_2_ (ergocalciferol) injection versus matching placebo every autumn over 3 years in 9440 men and women aged over 75 years found that femoral fractures were 49% higher and wrist fractures were 22% higher in the bolus‐treated compared to placebo‐treated participants.^(^ ^76^ ^)^ Another trial by Khaw and colleagues^(^ ^77^ ^)^ of 5108 largely replete older men and women (25(OH)D = 24 ng/mL) found that a starting D_3_ bolus of 200,000 IU followed by a monthly bolus doses of 100,000 IU did not prevent fractures or falls over 3 to 4 years. However, a study in older UK subjects, where median 25(OH)D averaged about 16 ng/mL, found that a less aggressive D_3_ bolus of 100,000 IU given every 4 months for 5 years reduced fractures by 22%.^(^ ^78^ ^)^

Some studies of daily dosing in relatively vitamin D replete groups of older adults have not demonstrated a major impact on nonvertebral fractures.^(^ ^73^, ^74^ ^)^ However, daily dosing has been more effective in older adults who are deficient. Most meta‐analyses have not utilized individual participant data that would allow control for age and sex, baseline and achieved levels of 25(OH)D, as well as daily versus bolus dosing, which are critical to the issue.^(^ ^59^ ^)^ Most recent meta‐analyses simply rejected the influence of vitamin D on fracture risk, but two conceded that vitamin D plus calcium reduced the risk of femoral fractures by 15%^(^ ^79^ ^)^ and up to 30%.^(^ ^80^ ^)^ Only one meta‐analysis has used participant data (31,022 from 11 trials of which 90% were female) from double‐blind RCTs only that are so essential to analyses.^(^ ^81^ ^)^ It showed that with a median dose of 800 IU vitamin D per day, older adults at risk of vitamin D deficiency had a 30% lower risk for hip fractures. The sensitivity analysis showed that the inclusion of studies with bolus dosing attenuated that benefit.^(^ ^81^ ^)^ The detrimental effect of bolus dosing with regard to fracture risk is evident in the recent meta‐analyses where trials using bolus dosing constituted two‐thirds of the weighting.^(^ ^57^, ^58^ ^)^

## Effects of Bolus Dosing on any Cancer and Cancer‐Related Mortality

An association between vitamin D deficiency and cancer has been observed for several decades and there are numerous mechanistic studies detailing how vitamin D and its analogs can influence cancer cells and reduce proliferation.^(^ ^82^, ^83^, ^84^, ^85^, ^86^, ^87^, ^88^, ^89^, ^90^, ^91^, ^92^ ^)^ However, results from clinical trials testing supplemental vitamin D were mixed, with an overall suggestion that vitamin D may have no benefit on the prevention of any cancer,^(^ ^93^, ^94^, ^95^ ^)^ but may reduce the risk of advanced cancer and fatal cancer.^(^ ^93^, ^94^, ^95^ ^)^ Consistently, a recent umbrella meta‐analysis of clinical trials of vitamin D supplementation found no benefit on cancer incidence, but a reduction of total cancer mortality risk, with five out of six meta‐analyses reporting a relative risk (RR) reduction of up to 16%.^(^ ^96^ ^)^ The large VITAL trial examined the effects of daily vitamin D supplements (2000 IU) on cancer and cardiovascular disease in 25,871 subjects over 5 years. Although the study did not demonstrate an effect on the incidence of any cancer,^(^ ^97^ ^)^ a detailed secondary analysis showed that non‐obese subjects had a 38% reduction of fatal and metastatic cancer.^(^ ^93^ ^)^ Also, VITAL found a 20% to 25% reduction in cancer mortality if the first or also second year of latency were excluded.^(^ ^97^ ^)^

Notably, in a cost‐benefit analysis, Niedermaier and colleagues^(^ ^98^ ^)^ calculated that a 13% lower cancer mortality through D_3_ supplementation of adults over age 50 years would prevent 30,000 deaths per year and result in net savings of $300 million in Germany alone.

With regard to a comparison of daily versus bolus dosing, a 2019 meta‐analysis identified 10 trials that tested vitamin D for cancer incidence and mortality, including the VITAL trial.^(^ ^95^ ^)^ The meta‐analysis had 6537 cases, follow‐ups were between 3 and 10 years, and 25(OH)D levels achieved were between 21 and 54 ng/mL in the intervention groups. Although there was no benefit of D_3_ on cancer incidence, the meta‐analysis documented a 13% reduction in cancer mortality with daily dosing of D_3_, but there was no benefit with bolus dosing.^(^ ^95^ ^)^ A case example is the large ViDA study of 5108 subjects that used a monthly bolus of 100,000 IU for up to 4 years and found no benefit for either cancer incidence or cancer mortality.^(^ ^99^ ^)^

## Summary

In this review we summarized and discussed growing evidence that large bolus dosing of vitamin D may have minimal benefit, or even be counterproductive, whereas small to moderate daily dosing in individuals at risk of deficiency is beneficial. This applied to outcomes of rickets, musculoskeletal health (falls and fractures), as well as respiratory infections and cancer mortality, and possibly weekly dosing for calcifediol with regard to COVID‐19. However, as discussed above in the section on falls and fractures, the benefits of daily dosing have been absent in several studies among vitamin D–replete adults and those not at risk for the outcomes of interest (ie, falls and factures^(^ ^73^, ^74^ ^)^), and although this needs further study, we cannot exclude that higher daily doses may also trigger countervailing factors.^(^ ^24^ ^)^

The increased use of bolus dosing in a growing number of trials may in part be motivated by convenience and purported benefits on adherence compared to daily dosing.^(^ ^100^ ^)^ In addition, the frequent use of bolus dosing for trials of vitamin D may be due to a misinterpretation of its short‐term increase in 25(OH)D levels, whereas longer‐term countervailing factors triggered by such doses that turn off its tightly regulated hormonal activation process have been overlooked.^(^ ^3^ ^)^ The obvious countervailing increase of 24‐hydroxylase leads to downregulation of 1,25(OH)_2_D and inhibits immune‐modulation for weeks or even months.^(^ ^3^ ^)^ In other words, although a large bolus produces a quick increase in 25(OH)D levels, it does so at the cost of downregulation of cellular activation and of factors of immunity.^(^ ^101^ ^)^ In contrast, a small to moderate dose of daily D_3_ has superior intracellular effects and needs frequent dosing due to its 20‐hour half‐life.^(^ ^3^, ^11^, ^24^ ^)^ If a rapid increase of 25(OH)D is needed, administration of an initial low calcifediol dose may be an alternative.^(^ ^50^, ^51^ ^)^ And once the 25(OH)D level is up, calcifediol would no longer be needed.

It has been difficult to isolate the influence of bolus dosing in meta‐analyses because many authors have merged these trials with those using daily dosing. Further, the low cost and high safety of D_3_ has led to studies in the broad public, not just in deficient persons or those at clinical risk, so the usual criteria of clinical trials may not be appropriate. Therefore, public health agencies are challenged to make a comprehensive risk‐benefit assessment of the potential role of a daily small to moderate dose of vitamin D in the current COVID pandemic, particularly for those with known deficiency (older adults and darker‐skinned ethnic groups).^(^ ^8^, ^9^ ^)^ Notably, even a decade ago it became apparent that bolus dosing was “too much of a good thing,”^(^ ^102^ ^)^ whereas correction of existing deficiencies with daily dosing of vitamin D is a low‐cost and safe public health strategy in ameliorating a host of affected disorders.

### Peer Review

The peer review history for this article is available at https://publons.com/publon/10.1002/jbm4.10567.
